# Evaluation of the Effects of the Anti-Inflammatory and Antioxidant Properties of Aloperine on Recovery in an Experimental Sciatic Nerve Injury Model

**DOI:** 10.3390/antiox15010126

**Published:** 2026-01-19

**Authors:** Mehmet Ertanıdır, Erkan Sabri Ertaş, Ali Güleç, Bahadır Öztürk, Nejat Ünlükal, Sadettin Çiftci

**Affiliations:** 1 Department of Orthopaedics and Traumatology, Faculty of Medicine, Selçuk University, Konya 42130, Türkiye; erkansabri@yahoo.com (E.S.E.); dr.aligulec@selcuk.edu.tr (A.G.); 2 Department of Biochemistry, Faculty of Medicine, Selçuk University, Konya 42130, Türkiye; bahadirozturk@selcuk.edu.tr; 3 Department of Histology and Embryology, Faculty of Medicine, Selçuk University, Konya 42130, Türkiye; nunlukal@selcuk.edu.tr

**Keywords:** aloperine, peripheral nerve healing, antioxidant, anti-inflammatory, rat

## Abstract

Peripheral nerve injuries affect 13–23 out of 100,000 people annually, with Wallerian degeneration and subsequent inflammatory/oxidative responses critically impacting recovery. Aloperine, a natural alkaloid from *Sophora alopecuroides* L., exhibits potent anti-inflammatory and antioxidant properties but has never been studied for nerve repair. In this study, we aimed to investigate whether aloperine could enhance peripheral nerve regeneration by modulating inflammation and oxidative stress in a rat sciatic nerve injury model. Thirty male Wistar rats underwent sciatic nerve neurotmesis with epineural repair. Animals were divided into surgical controls (Group A), aloperine-treated rats (Group B; single 100 mg/kg intraperitoneal dose), and intact controls (Group C). After 8 weeks, outcomes were assessed via functional tests (pinprick, hot plate, extensor postural thrust), biochemical analyses (TNF-α, IL-6, IL-10, TOS/TAS), and histomorphometric evaluations (axon counts, diameter indices, immunohistochemistry). Aloperine treatment significantly improved functional recovery, with near-normal hot plate latency and motor performance. Biochemically, it reduced pro-inflammatory markers (TNF-α) while elevating IL-10. Oxidative stress was attenuated. Histologically, treated nerves showed better-preserved axonal architecture (reduced inflammation). This first investigation of aloperine for nerve repair demonstrates its therapeutic potential through dual anti-inflammatory and antioxidant mechanisms, significantly improving functional and structural outcomes. These findings support its development as a novel treatment for peripheral nerve injuries.

## 1. Introduction

Peripheral nerve injuries are common traumatic events, with an incidence of 13–23 per 100,000 in developed countries [1]. When a nerve fiber is injured, chemical, functional, and morphological changes occur in the nerve, collectively referred to as Wallerian degeneration [2]. Immune cells such as neutrophils, T lymphocytes, and macrophages contribute to the pathogenic effects observed in Wallerian degeneration [3]. Following injury, the immune system is activated, leading to increased release of immune mediators and heightened oxidative stress, which in turn adversely affects Wallerian degeneration [4]. Aloperine is a quinolizidine alkaloid with well-documented antioxidant, anti-inflammatory, antibacterial, antiviral, and antitumoral properties [5]. Findings from recent studies have indicated that aloperine exerts neuroprotective effects in various central nervous system injury models by reducing oxidative stress, modulating inflammatory cytokines, and suppressing apoptosis [6]. Experimental data indicate that its biological activity is observed at doses between 50 and 100 mg/kg, which have been reported as effective and safe in vivo with meaningful pharmacological effects demonstrated even after single-dose administration [7,8]. Considering these established central neuroprotective mechanisms, it is reasonable to hypothesize that aloperine may also provide beneficial effects in the peripheral nervous system, particularly during the inflammatory and oxidative phases of Wallerian degeneration.

Neurometastasis injuries represent a common form of peripheral nerve damage and have, therefore, been extensively investigated using experimental models. Their widespread use is primarily based on their ability to closely mimic mechanical nerve injuries frequently observed in real-life clinical settings [9]. In this context, *Wistar Albino* rats are a well-known model due to their standardized sciatic nerve anatomy, reproducible functional outcomes, and strong similarity to humans in the pathophysiology of Wallerian degeneration. Furthermore, previous experimental studies have demonstrated the beneficial effects of aloperine on the central nervous system, providing further justification for the use of an experimental animal model to investigate its potential role in peripheral nerve regeneration.

The aim of this study was to investigate the effects of aloperine, which has documented antioxidant and anti-inflammatory activity, on peripheral nerve healing, using histological, functional, and biochemical evaluations.

## 2. Materials and Methods

The study commenced after ethical approval was obtained from the Local Animal Experiments Ethics Committee of the Selçuk University Experimental Medicine Application and Research Center (decision number 2022-29, dated 29 July 2022). Thirty 12–16 week-old male Wistar Albino rats, each weighing 350–400 g, were included in the study. Prior to and following surgery, the rats were provided ad libitum access to food and water. After surgery, the animals were housed individually in separate cages.

### 2.1. Groups

A total of 30 male Wistar Albino rats were randomized into three groups using block randomization: two surgical groups and one control group. In the surgical groups, a neurotmesis injury was created in the right sciatic nerve, followed by epineural repair. A 12 h light/dark cycle was maintained, along with room temperature and free access to dry feed pellets and water. The groups were as follows:

Group A: Surgery was performed; no aloperine was administered.
Group B: Surgery was performed; a single intraperitoneal dose of aloperine was administered.
Group C (Control): No surgical intervention performed.

### 2.2. Surgical Procedures

Anesthesia was induced via intraperitoneal injection of Ketamine Hydrochloride (Ketalar^®^ vial, Eczacıbaşı Pharmaceuticals and Industrial Company, Istanbul, Turkey; 50 mg/kg) and Xylazine (Rhompun^®^ injectable vial, Bayer AG, Leverkusen, Germany; 10 mg/kg). All procedures were performed on the right sciatic nerve. Rats were positioned prone on the surgical table. A line connecting the right knee to the right ischial tuberosity was marked with a surgical pen. After surgical site preparation with 10% povidone-iodine, the area was draped in a sterile fashion.

**Figure 1:**
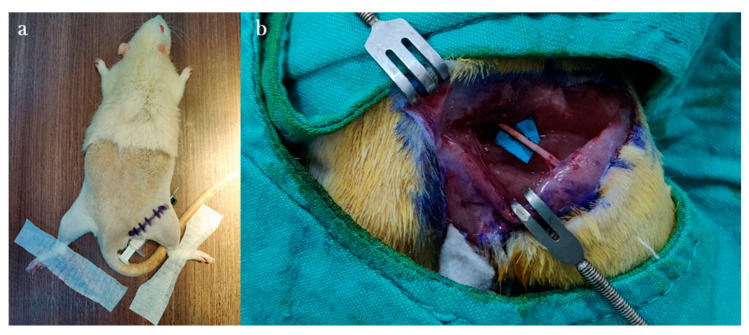
Preparation of rats on the platform before the surgical procedure (**a**) and anatomical view after rat sciatic nerve exploration (**b**).

A 3 cm incision was made along the marked line, parallel to the femur. Following skin dissection, the interval between the gluteus and biceps femoris muscles was accessed. After visualizing the vastus lateralis, the biceps femoris was retracted posteriorly to expose the sciatic nerve. The transection site was determined to be 1 cm proximal to the trifurcation of the sciatic nerve. Under microscopic visualization, the nerve was completely transected in a single motion using a No. 11 scalpel blade to create the neurotmesis model (Figure 1).

**Figure 2:**
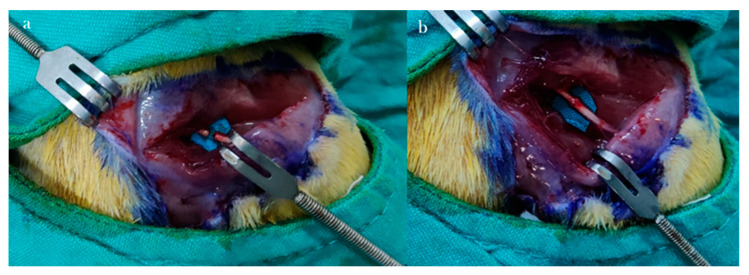
Neurometosis type injury in the sciatic nerve (**a**) and image after epineural coaptation (**b**).

Epineural coaptation was then performed under a surgical microscope (World Precision Instruments; Sarasota, FL, USA) using 9-0 monofilament nylon sutures (Ethilon, Ethicon; Somerville, NJ, USA). Four sutures were placed for end-to-end epineurial repair. All repairs were performed by a single experienced hand surgeon. Care was taken to align fascicles appropriately. After repair, the surgical site was irrigated with sterile isotonic saline, the muscle fascia was closed with 4-0 Vicryl sutures, and the skin was closed with 4-0 Prolene sutures (Figure 2). No antibiotic prophylaxis was administered, in accordance with institutional guidelines for clean surgical procedures.

In the aloperine group, the animals received a single intraperitoneal dose of aloperine (Abcam, Aloperine, Monmouth Junction, NJ, USA, >99% purity) at 100 mg/kg, dissolved in 2 mL of physiological saline, administered after completion of the surgical procedure. After recovery from anesthesia, rats were placed in separate cages, for analgesic management, paracetamol (2 mg/mL) was added to the drinking water for 7 days postoperatively. Animals underwent regular wound monitoring, and no postoperative complications were observed throughout the study period. No animal deaths or exclusions occurred during the study. All rats completed the experimental protocol as planned, and no animals were removed from the analysis. Following the 8-week observation period, functional tests were performed, after which the animals were sacrificed under high-dose anesthesia.

Through the previous incision line, the sciatic nerve was re-exposed, and the segment including 2 cm proximal and distal to the repair site was excised, with marking sutures placed at both ends. Both gastrocnemius muscles were also removed for analysis.

The surgeon performing the sciatic nerve injury and treatment procedures was blinded to group assignments. The personnel administering aloperine or vehicle solutions prepared coded syringes, and the surgeon did not know which animals received aloperine.

### 2.3. Functional Evaluation

Before sacrifice, all rats underwent pinprick, hot plate, and the extensor postural thrust (EPT) testing. These functional assessments were performed only at this final time point.

#### 2.3.1. Pinprick Test

**Figure 3:**
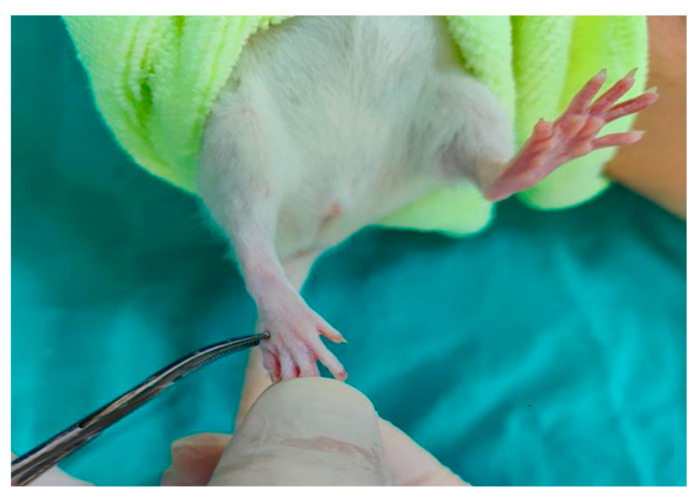
Pinprick Test.

The skin from the foot to the knee was compressed with the same forceps to elicit a pain withdrawal reflex. A response at the metatarsal level was scored as 3 points, at the heel or distal ankle level as 2 points, and at the proximal ankle level as 1 point. Absence of reflex response was scored as 0 [10] (Figure 3).

#### 2.3.2. Extensor Postural Thrust Test

**Figure 4:**
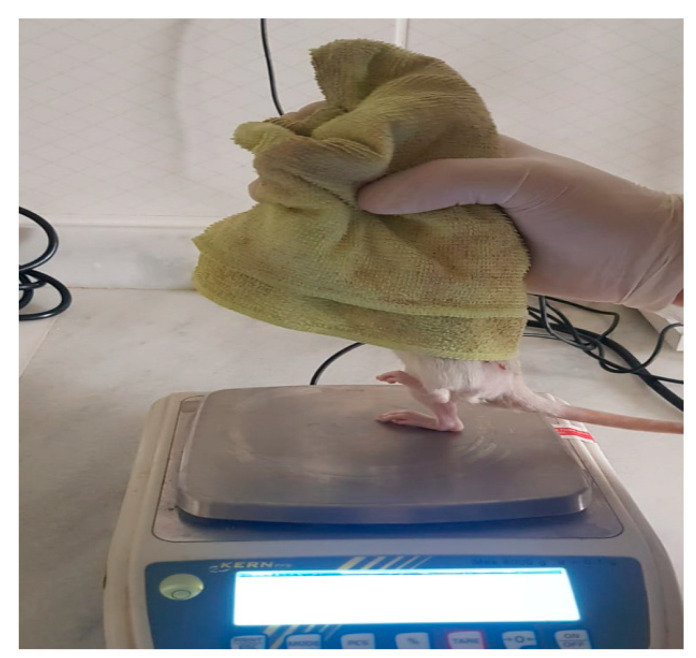
Extensor postural thrust test. The procedure was repeated five times, and the highest value was recorded.

This test was used to evaluate motor performance. The operated foot was placed on a precision scale, and the force exerted was measured. The procedure was repeated five times, and the highest value was recorded. The same procedure was performed for the contralateral limb. Data were analyzed using the thrust ratio formula described by Koka and Hadlock [1] (Figure 4).

#### 2.3.3. Hot Plate Test

**Figure 5:**
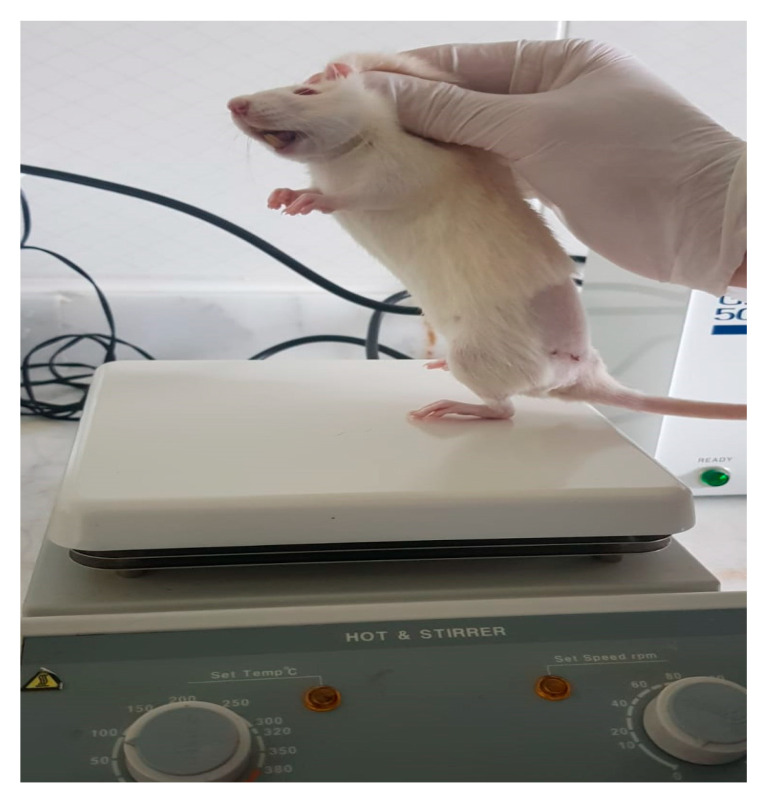
Hot plate test. The maximum cut-off time was 12 s.

This assessed thermal sensation. The lateral aspect of the operated paw was placed on a surface preheated to 56 °C, and the withdrawal latency was recorded using a stopwatch. The test was repeated three times at 2 min intervals, and the mean latency was calculated. The maximum cut-off time was 12 s to prevent burns [2] (Figure 5).

#### 2.3.4. Gastrocnemius Muscle Weight Index

**Figure 6:**
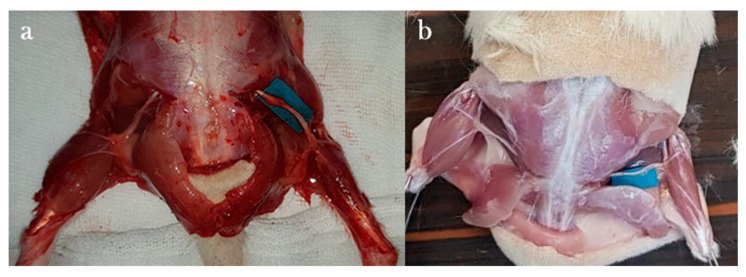
Difference in volume between gastrocnemius: (**a**) Group A; (**b**) Group B.

Bilateral gastrocnemius muscles were removed postmortem and weighed on a precision balance. The index was calculated by dividing the weight of the operated-side muscle by that of the contralateral side [3] (Figure 6).

### 2.4. Biochemical Evaluation

Blood samples were collected using yellow-cap, gel-containing serum separator tubes and centrifuged at 3500 rpm for 10 min. The obtained serum was aliquoted into Eppendorf tubes and stored at −80 °C until analysis. On the day of analysis, samples were thawed and evaluated using rat ELISA kits for TNF-α (BT Lab-Biotech Co., Ltd., Shanghai, China; catalog no. E0764Ra), IL-6 (catalog no. E0135Ra), and IL-10 (catalog no. E0108Ra), according to the manufacturer’s protocols. Absorbance was measured at 450 nm with a CLARIO star microplate reader (BMG LABTECH GmbH, Ortenberg, Germany).

Serum total oxidant status (TOS) and total antioxidant status (TAS) were measured using commercial kits (Rel Assay Diagnostics, Gaziantep, Turkey; TAS Lot: OK22136A, TOS Lot: OK22150O) on a Roche Cobas c 501 autoanalyzer (Ibaraki, Japan) via photometric methods.

### 2.5. Histological and Immunohistochemical Evaluation

For histological analysis, tissues were fixed in freshly prepared 4% paraformaldehyde at +4 °C for 24 h, then transferred to 30% sucrose until the tissue sank (minimum 24 h). Samples were embedded in cryomatrix and sectioned at 4 μm thickness using a cryostat (Thermo Shandon Cryostat 210160GB, Thermo Fisher Scientific, Waltham, MA, USA). Hematoxylin and eosin (H&E) staining was performed for evaluation of inflammation and general histology. Toluidine blue staining was performed by dissolving 0.1 g of dye in 100 mL of distilled water; sections were stained for 1–2 min, rinsed, and coverslipped.

Proximal and distal axon counts were determined using NIS-Elements software, version 5.21 (Nikon Instruments Inc., Tokyo, Japan). The axon number change index (ANCI) was calculated by dividing the distal axon count by the proximal count. Axon diameter measurements were taken in randomly selected fields, and the axon diameter change index (ADCI) was calculated as the distal-to-proximal mean axon diameter ratio.

Immunofluorescence staining was performed on the proximal segments of the sciatic nerve. Primary antibodies against TNF-α and IL-10 were used at a 1:200 dilution. Staining was carried out under dark, humid conditions, slides were examined with an Olympus BX51 trinocular fluorescence microscope (Olympus Corporation, Tokyo, Japan), and four random fields per sample were photographed at 40× magnification (DP72 camera, Olympus Corporation, Tokyo, Japan). TNF-α (FITC filter, Olympus Corporation, Tokyo, Japan, green fluorescence) was used as the pro-inflammatory marker and IL-10 (TXRED filter, Olympus Corporation, Tokyo, Japan, red fluorescence) as the anti-inflammatory marker. Staining intensity was scored from 0 to 3.

All histological and biochemical assessments were performed by investigators who were fully blinded to the group identities. Tissue samples were labeled with anonymized numeric codes until all analyses were completed.

### 2.6. Statistical Analysis

All statistical analyses were carried out using Python (version 3.12) with standard scientific libraries, including SciPy, NumPy, and Statsmodels. Because several outcome variables did not satisfy the assumptions required for parametric testing, group comparisons were performed using the Kruskal–Wallis test. When the overall test indicated a significant group effect, pairwise comparisons were conducted using the Mann–Whitney U test. Effect sizes for pairwise tests were calculated using the formula r = Z/√N.

To account for multiple testing, two correction procedures were applied: the Holm step-down method and the Benjamini–Hochberg false discovery rate (FDR). Statistical significance was defined as *p* < 0.05.

## 3. Results

The Kruskal–Wallis test showed significant group differences for extensor postural thrust ratio (H = 24.27, *p* < 0.001), hot plate latency (H = 25.35, *p* < 0.001), gastrocnemius muscle index (H = 21.77, *p* < 0.001), serum TNF-α (H = 10.45, *p* = 0.0054), serum IL-6 (H = 9.09, *p* = 0.0106), serum IL-10 (H = 25.30, *p* < 0.001), serum TAS (H = 6.92, *p* = 0.0314), axon number change index (H = 7.21, *p* = 0.0272), and axon diameter change index (H = 25.95, *p* < 0.001). Serum TOS values did not differ significantly among the groups (H = 5.67, *p* = 0.0587). Pinprick scores also did not show a significant group effect (H = 5.12, *p* = 0.0773).

### 3.1. Functional Evaluation Results

For extensor postural thrust ratio, all pairwise group comparisons were significant after Holm adjustment (*p* ≤ 0.0011), and effect sizes were large across all contrasts (r = 0.74–0.85). Hot plate latency showed a similar pattern, with all pairwise comparisons remaining significant after Holm correction (*p* ≤ 0.00052), again accompanied by large effect sizes (r = 0.81–0.85).

**Figure 7:**
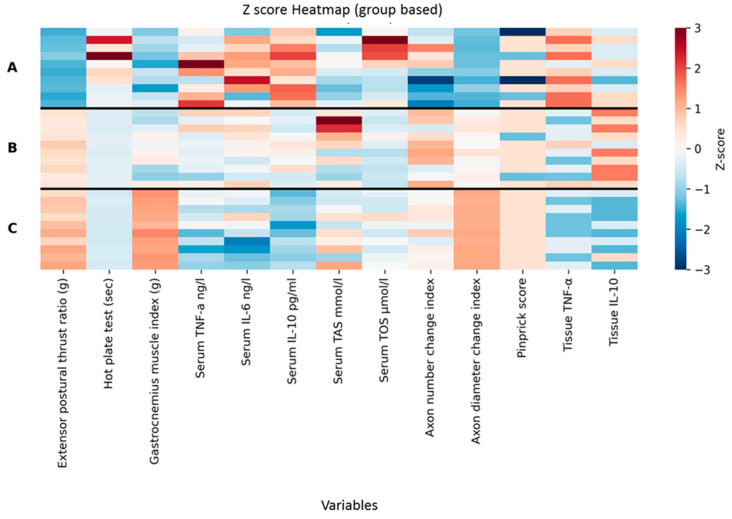
Z-score–normalized heatmap of group-wise distributions across all study variables. Each row represents an individual subject, grouped by experimental condition (A, B, and C), with horizontal black lines indicating boundaries between groups. Columns correspond to the analyzed variables. Heatmap colors represent Z-score–normalized values calculated separately for each variable using the formula Z = (X − Y)/Z, where X is the raw observed value, Y is the mean, and Z is the standard deviation of that variable across all subjects. The color scale is centered at zero, with red tones indicating values above the overall mean and blue tones indicating values below the mean. Z-scores were clipped to the range of −3 to +3 to enhance visual contrast. Raw (non-normalized) data were used for all statistical analyses; Z-score normalization was applied solely for visualization purposes.

Gastrocnemius muscle index also demonstrated significant differences for all three pairwise comparisons (p_Holm = 0.0233), with effect sizes ranging from moderate to large. Serum IL-10 followed the same pattern, with all pairwise contrasts significant after adjustment (*p* ≤ 0.00055), each associated with large effect sizes. The results are presented in Table 1 and Figure 7.

### 3.2. Biochemical Evaluation Results

For serum TNF-α, serum IL-6, and serum TAS, although the Kruskal–Wallis test indicated significant overall differences, none of the pairwise comparisons reached significance after Holm correction. Additional contrasts became significant under the FDR procedure: for TNF-α, the comparison between groups A and C (p_FDR = 0.0108), and for IL-6, the comparisons A vs. C (p_FDR = 0.0386) and B vs. C (p_FDR = 0.0174). No FDR-significant contrasts were observed for TAS.

### 3.3. Histological and Immunohistochemical Evaluation Results

**Figure 8:**
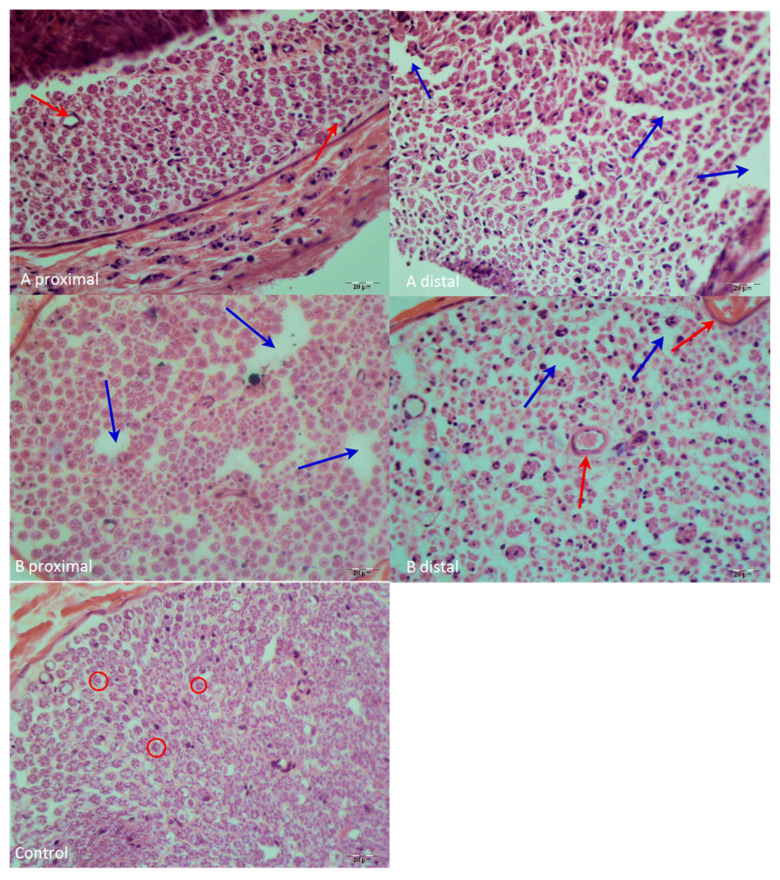
Microscopic view of the groups stained with hematoxylin and eosin at 40× magnification. H&E-stained sections demonstrating distal and proximal nerve morphology in Groups A, B, and the control. Group A shows marked axonal degeneration and widened gaps, particularly in distal segments. Group B exhibits better-preserved axonal architecture with fewer gaps and morphology closer to the control group. Control sections display normal nerve structure without degenerative changes. Red arrows indicate blood vessels, blue arrows indicate vacuolar spaces associated with tissue damage, and red circles indicate axons.

Hematoxylin & Eosin (H&E) staining findings: In the distal segment of the repair site in Group A, axonal architecture was disrupted with large gaps caused by axonal shrinkage. Schwann cell nuclei exhibited pyknotic changes. These changes, along with shrinkage and gaps, were more prominent in the peripheral regions of the nerve, whereas the central portion was relatively better preserved. No necrotic areas were observed, but inflammatory findings were present. In the proximal segment, axonal architecture appeared better preserved than distally, with occasional gaps, and the central–peripheral transition zone showed relatively better morphology (Figure 8).

In Group B, distal to the repair site, occasional gaps were present in the nerve periphery, though less pronounced than in Group A. The central portion was better preserved, with more condensed nuclei in the periphery. Although axonal architecture was disrupted, it was in better condition than Group A, and increased capillary density was observed. No necrotic areas were present, and inflammatory findings were minimal, comparable to Group C. Proximally, axonal architecture was less disrupted compared to distally, with gaps due to axonal shrinkage and pyknotic Schwann cell nuclei observed, though nearly similar to the control group. When comparing the axon diameter change index and axon number change index, Group B showed significantly higher values than Group A (*p* < 0.05), with no significant difference compared to Group C (*p* > 0.05).

Axon number change index showed a statistically significant overall difference; however, none of the pairwise comparisons remained significant following Holm or FDR adjustment. In contrast, the axon diameter change index demonstrated significant differences between all group pairs after Holm correction (*p* ≤ 0.00048), with effect sizes consistently in the very large range (r ≈ 0.85).

**Figure 9:**
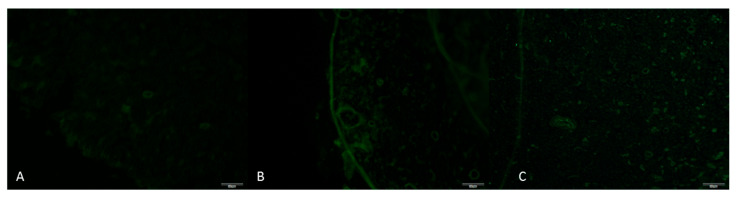
Immunofluorescence TNF-α staining image of sciatic nerves of rats at 40× magnification. (**A**) Group A; (**B**) Group B; (**C**) Group C. Immunofluorescence images demonstrate TNF-α staining patterns for the experimental groups. Green fluorescence corresponds to TNF-α–positive immunoreactivity (FITC filter) (Scale bar = 20 µm).

**Figure 10:**
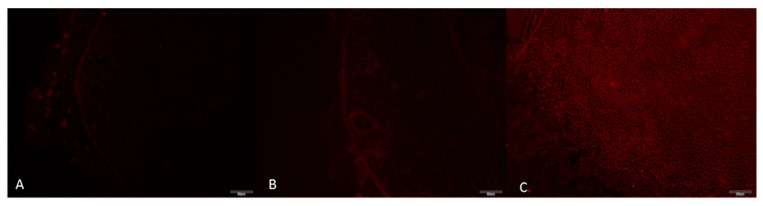
Immunofluorescence IL-10 staining image of sciatic nerves of rats at 40× magnification. (**A**) Group A, (**B**) Group B, (**C**) Group C. Immunofluorescence images demonstrate IL-10 staining patterns for the experimental groups. Red fluorescence corresponds to IL-10 positive immunoreactivity (FITC filter) (Scale bar = 20 µm).

Among ordinal variables, significant overall differences were found for tissue TNF-α and tissue IL-10 (both *p* = 0.0002). For tissue IL-10, all pairwise comparisons were significant after Holm adjustment (*p* = 0.024), with effect sizes between 0.48 and 0.77. For tissue TNF-α, no pairwise comparisons reached significance under Holm correction, whereas the FDR approach identified significant differences between groups A and B (p_FDR = 0.00196) and between groups A and C (p_FDR = 0.00072), both associated with large effect sizes (Figure 9 and Figure 10).

A detailed summary of all statistical outcomes, including test statistics, pairwise *p*-values (raw, Holm-adjusted, and FDR-adjusted), and effect sizes, is presented in Table 1 and Figure 7.

## 4. Discussion

Peripheral nerve regeneration is a complex process that requires a delicate balance between inflammation and oxidative stress [4,11]. While inflammation is necessary for initiating repair, excessive or prolonged inflammatory responses can hinder recovery [10,12]. Similarly, oxidative stress exacerbates tissue damage and delays functional regeneration [13,14]. In this study, we evaluated the effects of aloperine, a compound with known anti-inflammatory and antioxidant properties, on peripheral nerve healing. Our findings demonstrate that aloperine significantly improves functional, biochemical, and histological outcomes, offering new insights into its therapeutic potential for nerve repair.

Functional recovery serves as one of the most reliable indicators of successful nerve regeneration, reflecting the restoration of axonal integrity, remyelination, and neuromuscular connectivity [15,16]. In the present study, aloperine administration led to marked improvements in both sensory and motor functions compared to the control group. These results are consistent with previous research showing that functional tests reliably reflect the degree of axonal regeneration and target reinnervation, while also serving as a more clinically relevant measure of nerve repair success than histological evaluation alone [17,18]. Although the pinprick test did not reveal a statistically significant difference between groups, this may be attributed to the limited sensitivity of this test and its susceptibility to ceiling effects at later stages of recovery. In contrast, the extensor postural thrust, muscle weight index, and hot plate tests—representing more quantitative and integrative functional assessments—demonstrated significant improvements. The observed enhancements in aloperine-treated animals suggest that this compound may facilitate faster and more complete functional restoration following nerve injury.

The inflammatory response following nerve injury is tightly regulated by the interplay of pro- and anti-inflammatory cytokines [19,20]. In the inflammatory cascade, the main pro-inflammatory cytokines are TNF-α and IL-6, while IL-10 is an anti-inflammatory cytokine [21]. In our study, aloperine treatment resulted in decreased levels of pro-inflammatory cytokines (TNF-α and IL-6) alongside increased levels of the anti-inflammatory cytokine IL-10. These findings align with research demonstrating the anti-inflammatory effects of aloperine in other pathological conditions [22,23]. The reduction in pro-inflammatory markers is particularly significant, as studies have shown that excessive levels of these cytokines are linked to delayed regeneration [24,25]. Conversely, the elevation of IL-10, which has been reported to support axonal growth [26], further underscores aloperine’s potential benefits in nerve repair.

Oxidative stress represents another critical factor that negatively impacts nerve regeneration [13,27]. In this study, aloperin treatment resulted in a significant increase in serum TAS levels, indicating increased systemic antioxidant capacity, while serum TOS levels were lower in the group treated with aloperin, although this difference was not statistically significant. This can be attributed to the timing of the assessment, as the oxidative load is generally more pronounced in the early post-injury period and returns to normal by week 8. In contrast, the total antioxidant status reflects a more sustainable adaptive response, suggesting that aloperin may contribute to long-term redox balance rather than acute suppression of oxidant production. These findings are consistent with previous studies reporting that the antioxidant effects of aloperin occur primarily through the enhancement of endogenous antioxidant defense mechanisms [6,28]. Studies have indicated that oxidative damage disrupts the regenerative microenvironment [27,29]. When these findings are considered together, it suggests that aloperin may reduce oxidative load in the acute phase after nerve injury, and in later stages of healing, it may primarily increase total antioxidant capacity, thus positively contributing to nerve recovery.

Histopathological examination revealed that aloperine-treated nerves exhibited better-preserved axonal architecture, increased vascularity, and reduced inflammatory infiltration compared to controls. These morphological improvements are consistent with the functional and biochemical outcomes observed in our study. Previous studies have indicated that axonal number and diameter are key indicators of regenerative success [30,31]. The increased axon diameter and number in our aloperine-treated animals suggest enhanced axonal maturation and myelination, similar to findings in other investigations [32]. Additionally, the reduction in inflammatory cell infiltration supports observations that maintaining vascular integrity is crucial for nerve regeneration [33].

Aloperin has reportedly modulated several key pathways involved in inflammation and oxidative stress, including NF-κB, MAPK, and JAK/STAT signaling [34]. Suppression of NF-κB activation reduces the transcription of major pro-inflammatory mediators such as TNF-α and IL-6, while enhancement of IL-10 production supports anti-inflammatory processes. In addition, aloperin exhibits antioxidant properties by decreasing total oxidant status and improving the overall redox balance. Based on our results, it is plausible that aloperin promotes peripheral nerve regeneration by attenuating inflammatory responses, limiting oxidative damage at the injury site, and thereby facilitating axonal repair and tissue remodeling.

### Limitations

This study has several limitations that should be acknowledged. First, an electromyography (EMG) assessment could not be performed, which may have provided additional objective information regarding neuromuscular function. Second, although functional recovery was evaluated, a longer follow-up period for gait and walking assessments could have offered further insight into the long-term progression of nerve regeneration. Third, the study was conducted with a relatively limited number of animals, as the sample size was intentionally kept to the minimum required in accordance with ethical principles aimed at reducing animal use; studies with larger cohorts may provide greater statistical power. Finally, tissue-level oxidative stress markers (TAS and TOS) could not be measured directly in nerve tissue, as the mass of the rat sciatic nerve was insufficient for reliable biochemical analysis. Future studies addressing these limitations may help to further clarify the effects of aloperine on peripheral nerve regeneration.

## 5. Conclusions

In conclusion, our study provides the first evidence that aloperine enhances peripheral nerve regeneration through its anti-inflammatory and antioxidant properties. By modulating cytokine levels, reducing oxidative stress, and improving histological outcomes, aloperine promotes both structural and functional recovery after nerve injury. These findings highlight its potential as a therapeutic agent for peripheral nerve repair and warrant further investigation into its clinical applications.

## Figures and Tables

**Table 1:** Summary of histological, biochemical, and functional outcomes across all study groups.

Variable	Mean ± SD (Median, Min, Max)	KW H	*p* *	Pairwise Comparison	r	p_raw	p_Holm	p_FDR
Extensor postural thrust ratio (g)	A 0.71 ± 0.20 B 0.59 ± 0.01 C 0.06 ± 0.01	24.27	<0.001	A vs. B	0.845	0.00018	0.00113	0.00027
				A vs. C	0.845	0.00018	0.00113	0.00027
				B vs. C	0.735	0.00113	0.00113	0.00113
Hot plate test (s)	A 3.39 ± 0.61 B 1.07 ± 0.03 C 0.80 ± 0.02	25.35	<0.001	A vs. B	0.845	0.000174	0.000521	0.000274
				A vs. C	0.845	0.000183	0.000365	0.000274
				B vs. C	0.811	0.000315	0.000315	0.000315
Gastrocnemius muscle index (g)	A 0.44 ± 0.03 B 0.59 ± 0.03 C 0.95 ± 0.00	21.77	<0.001	A vs. B	0.516	0.02329	0.02329	0.02329
				A vs. C	0.845	0.00018	0.02329	0.00027
				B vs. C	0.845	0.00018	0.02329	0.00027
Serum TNF-α (ng/L)	A 256.31 ± 21.86 B 209.21 ± 8.93 C 175.69 ± 9.48	10.45	0.0054	A vs. B	0.372	0.10411	0.10411	0.10411
				A vs. C	0.659	0.00361	0.10411	0.01083
				B vs. C	0.456	0.04515	0.10411	0.06773
Serum IL-6 (ng/L)	A 5.06 ± 0.35 B 4.20 ± 0.17 C 3.51 ± 0.28	9.09	0.0106	A vs. B	0.270	0.24132	0.24132	0.24132
				A vs. C	0.507	0.02575	0.24132	0.03862
				B vs. C	0.625	0.00579	0.24132	0.01739
Serum IL-10 (pg/mL)	A 204.42 ± 2.78 B 233.58 ± 3.93 C 265.54 ± 4.77	25.30	<0.001	A vs. B	0.811	0.000330	0.000330	0.000330
				A vs. C	0.845	0.000183	0.000548	0.000274
				B vs. C	0.845	0.000183	0.000365	0.000274
Serum TAS (mmol/L)	A 1.26 ± 0.02 B 1.43 ± 0.05 C 1.36 ± 0.03	6.92	0.0314	A vs. B	0.541	0.01705	0.49597	0.05115
				A vs. C	0.439	0.05381	0.49597	0.08072
				B vs. C	0.161	0.49597	0.49597	0.49597
Serum TOS (µmol/L)	A 15.31± 2.49 B 4.98 ± 0.52 C 6.80 ± 0.59	5.67	0.0587	—	—	—	—	—
Axon number change index	A 0.79 ± 0.11 B 1.15 ± 0.05 C 1.02 ± 0.02	7.21	0.0272	A vs. B	0.524	0.02113	0.17530	0.06340
				A vs. C	0.347	0.12985	0.12985	0.12985
				B vs. C	0.389	0.08765	0.17530	0.12985
Axon diameter change index	A 0.90 ± 0.01 B 0.95 ± 0.01 C 0.99 ± 0.001	25.95	<0.001	A vs. B	0.845	0.000179	0.000179	0.000179
				A vs. C	0.845	0.000163	0.000326	0.000179
				B vs. C	0.845	0.000159	0.000478	0.000179
Pinprick score	A 2.00 ± 0.11 B 2.80 ± 1.33 C 3.00 ± 0.00	5.12	0.0773	—	—	—	—	—
Tissue TNF-α	A 2.4 ± 0.22 B 0.9 ± 0.23 C 0.5 ± 0.16	17.57	0.0002	A vs. B	0.701	0.001304	0.22385	0.00196
				A vs. C	0.803	0.000241	0.22385	0.000722
				B vs. C	0.254	0.22385	0.22385	0.22385
Tissue IL-10	A 1.3 ± 0.21 B 2.4 ± 0.22 C 0.5 ± 0.22	16.77	0.0002	A vs. B	0.600	0.005176	0.02403	0.00776
				A vs. C	0.482	0.02403	0.02403	0.02403
				B vs. C	0.769	0.000438	0.02403	0.00131

Values are presented as mean ± SD. Group A: surgery without aloperine; Group B: surgery with a single intraperitoneal dose of aloperine; Group C: control group without surgical intervention. * *p* < 0.05 indicates statistically significant difference among groups.

## Data Availability

All data generated as part of this study are included in the article.

## Abbreviations

The following abbreviations are used in this manuscript:

TNF-α	Tumor Necrosis Factor-alpha
IL-6	Interleukin-6
IL-10	Interleukin-10
TOS	Total Oxidant Status
TAS	Total Antioxidant Status
ELISA	Enzyme-Linked Immunosorbent Assay
